# Interferon-****α****-Induced Changes to Natural Killer Cells Are Associated with the Treatment Outcomes in Patients with HCV Infections

**DOI:** 10.1155/2013/374196

**Published:** 2013-08-13

**Authors:** Shinji Shimoda, Kosuke Sumida, Sho Iwasaka, Satomi Hisamoto, Hironori Tanimoto, Hideyuki Nomura, Kazufumi Dohmen, Kazuhiro Takahashi, Akira Kawano, Eiichi Ogawa, Norihiro Furusyo, Koichi Akashi, Jun Hayashi

**Affiliations:** ^1^Department of Medicine and Biosystemic Science, Graduate School of Medical Science, Kyushu University, Fukuoka 812-8252, Japan; ^2^The Center for Liver Disease, Shin-Kokura Hospital, Kitakyushu 803-8505, Japan; ^3^Department of Internal Medicine, Chihaya Hospital, Fukuoka 813-8501, Japan; ^4^Department of Medicine, Hamanomachi Hospital, Fukuoka 810-8539, Japan; ^5^Department of Medicine, Kitakyushu Municipal Medical Center, Kitakyushu 802-0077, Japan; ^6^Department of General Internal Medicine, Kyushu University Hospital, Fukuoka 812-8582, Japan

## Abstract

*Aim*. We analyzed the pretreatment natural killer (NK) cell functions with the aim of predicting the sustained virological response (SVR) or the interleukin (IL) 28B polymorphism that is strongly associated with the treatment response. *Methods*. The peripheral NK cells from chronic hepatitis patients with HCV genotype 1 and high virus titers were activated using a Toll-like receptor (TLR) 4 ligand and IFN-**α**. The cell surface markers were evaluated using a flow cytometric analysis, and IFN-**γ** production was evaluated using an enzyme-linked immunosorbent assay (ELISA). The genotyping of the polymorphisms in the *IL28B* gene region (rs8099917) on chromosome 19 was performed on the DNA collected from each patient. *Results*. The production of IFN-**γ** was significantly higher in the SVR patients compared with the no-response (NR) patients, whereas the cell surface markers were similar between the SVR and the NR patients. There were no significant differences found in the *IL28B* genotype distribution associated with the production of IFN-**γ**. *Conclusion*. Differences in the NK cell functions were observed between the SVR patients and the NR patients, suggesting that NK cells play a potential role in the treatment response independent of the *IL28B* genotype.

## 1. Introduction

The hepatitis C virus (HCV) is the major cause of chronic liver disease, with an estimated global prevalence of 2.5%, that is, 170 million people infected worldwide [1], and is a leading cause of cirrhosis, hepatocellular carcinoma, and liver transplantation [2]. Antiviral treatment, which is based on the combination of pegylated interferon- (IFN-) *α* and the nucleoside analog ribavirin (RBV), is associated with a sustained virologic response (SVR), that is, serum HCV RNA negatively for 6 months after the cessation of the antiviral therapy.

 The HCV genotype, viral load, age, and fibrosis stage are well known as pretreatment variables [3–6]; moreover, single nucleotide polymorphisms (SNPs) located in the region of the interleukin (IL) 28B gene have been strongly associated with an SVR [7, 8]. Although the *IL28B* genotype can be useful when making treatment decisions, this variable alone is not a perfect predictor of the treatment outcome.

The natural killer (NK) cells involved in innate immunity play central roles of defense against viral infections through a direct cytotoxic effect in the destruction of the virus-infected target cells and the production of inflammatory cytokines [9]. Furthermore, in contrast to T cells, NK cells do not require priming for the recognition of the target cells. There has been growing evidence that NK cells have an important role in mediating the IFN-induced viral clearance of chronic HCV infections when the cell surface markers or the cytotoxicity of NK cells is evaluated [10]. Furthermore, we previously demonstrated that activated NK cells have cytotoxity against autologous biliary epithelial cells in the presence of IFN-*α* [11].

We hypothesize that because NK cells rapidly produce IFN-*γ* using several cytokines including IFN-*α*, pretreated changes of NK cells in the presence of an *ex vivo* IFN-*α* stimulation would become an indicator for the virological response; likewise, insulin resistance is another pretreatment predictor as we previously reported [12]. Furthermore, since the *IL28B* gene has been strongly associated with an acute HCV clearance [13], the relationship between the *IL28B* genotype and the NK cell functions that play a central role in the early phase viral clearance was investigated.

## 2. Materials and Methods

### 2.1. Study Subjects

A total of 20 patients with a chronic HCV genotype 1 infection and high viral load (>5.0 log IU/mL) were studied. All of the patients were treatment-naïve prior to their enrollment. The study samples were collected prior to the start of a standard pegylated IFN-*α* and RBV therapy. The patients who had undetectable HCV RNA for at least 6 months after treatment were classified as sustained viral responders (SVR, *n* = 8); those who had undetectable HCV RNA in the end of the treatment, however, detectable after the treatment, were classified as partial responders (PR, *n* = 8), and those who had persistently detectable HCV RNA during the therapy were classified as null responders (NR, *n* = 4).

All of the subjects gave their written informed consent and the experimental protocols were conducted under the Guidelines of the Research Ethics Committee of Kyushu University.

### 2.2. *IL28B* Polymorphism Analysis

The single nucleotide polymorphism (SNP) testing of the *IL28B* gene (rs8099917) was completed for all of the patients using a real-time PCR method on the genomic DNA extracted from the whole blood samples. The heterozygotes (TGs) or the homozygotes (GGs) of the minor allele (G) were described as having the *IL28B* minor allele, whereas the homozygotes for the major allele (TT) were described as having the *IL28B* major allele [7].

### 2.3. Isolation of Peripheral Blood Mononuclear Cells and NK Cells

The peripheral blood mononuclear cells (PBMCs) were separated from the heparinized fresh blood using a Ficoll-Isopaque gradient centrifugation technique. The NK cells were negatively isolated from the PBMC using magnetic beads (NK isolation kit; Miltenyi Biotec, Auburn, CA, USA). A viability of >95% using a trypan blue dye exclusion technique and a purity of >90% using flow cytometry for CD56 positives were considered to be acceptable [11].

### 2.4. IFN-*γ* Production from NK Cells

In an effort to identify the nature of the cytokines that were involved in promoting the NK cell effector function, the supernatants from the NK cells that have been cultured for 2 days with or without pegylated IFN-*α* (3 *μ*g/mL, Schering-Plough, Kenilworth, NJ, USA) and lipopolysaccharide (LPS) (10 *μ*g/mL, Invitrogen, San Diego, CA, USA) were analyzed for the production of IFN-*γ*. Pegylated IFN-*α* instead of IFN-*α* was used as pegylated IFN-*α* working *in vivo* during the standard pegylated IFN-*α* and RBV therapy. The assays were performed using a sandwich ELISA (R&D Systems, Minneapolis, MN, USA) with a combination of unlabeled and biotin-coupled or enzyme-coupled monoclonal antibodies to each cytokine. Both of the activated and the resting NK cells were evaluated for cell surface markers. 

### 2.5. Flow Cytometric Analysis of the Cell Surface Antigens

In order to evaluate the cell surface antigen expression in the PBMC and NK cells (1 × 10^6^), the cells were stained at 4°C in the dark for 30 min, washed twice in 2 mL of a phosphate-buffered saline containing 1% bovine serum albumin and 0.01% sodium azide, and were fixed in 500 *μ*L of 1% paraformaldehyde. The cells were stained for CD3, CD16, and CD56 (BD Biosciences, San Diego, CA, USA). A two-color flow cytometry was performed using an FACSnCalibur Flow Cytometer (BD Biosciences).

### 2.6. Statistical Analysis

All of the experiments were performed in triplicate and the data points shown are the mean values of the results of these triplicates. The comparisons between the points for certain data sets were expressed as the mean ± standard deviation (SD), and the significance of differences was determined by Student's *t*-test. All of the analyses were 2-tailed and the *P* values <0.05 were considered to be significant. The statistical analyses were performed using the Intercooled Stata 8.0 software program (StataCorp, College Station, TX, USA).

## 3. Results

### 3.1. Subjects

The characteristics of patients who were undergoing the standard pegylated IFN-*α* and RBV combined therapy are summarized in Table 1. Patients who experienced a relapse following the administration of the standard therapy (partial responders) were excluded from this study. As expected, the *IL28B* TT genotype was dominant in the SVR patients (75%) and not dominant in the PR patients (50%) or the NR patients (50%).

### 3.2. Phenotypes and IFN-*γ* Production in NK Cells before and after IFN-*α* Stimulation

**Figure 1:**
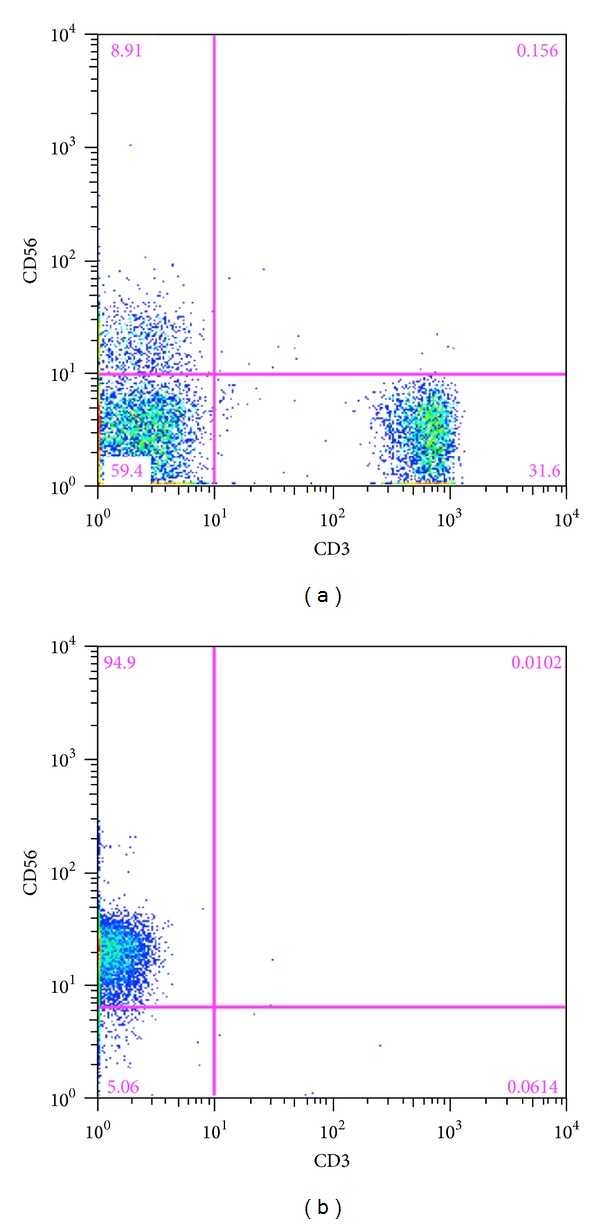
The isolation of the NK cells. The cell surface markers were determined in the PBMC using flow cytometry. (a) The PBMC constituted of CD3+CD56− cells (31.6%), CD3−CD56− cells (59.4%), CD3−CD56+ cells (8.91%), and CD3+CD56+ cells (0.2%). (b) Following the NK cell isolation, the number of CD3 positive cells had clearly decreased.

**Figure 2:**
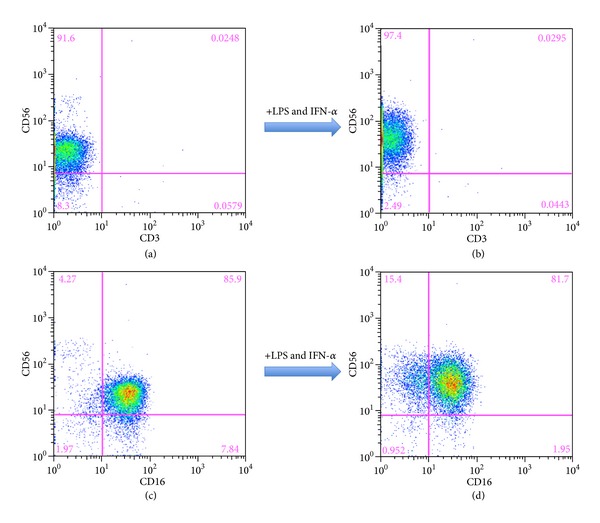
The surface markers of the NK cells before and after stimulation. The isolated NK cells were rested or stimulated with LPS and IFN-*α* for 2 days. Between (a) and (b), the CD3 and CD56 markers on the NK cells were not changed after stimulation. At (c), the resting NK cells were characterized as CD56+CD16− cells (4.3%), CD56+CD16+ cells (85.9%), and CD56−CD16+ cells (7.8%). At (d), the stimulated NK cells were characterized as CD56+CD16− cells (15.4%), CD56+CD16+ cells (81.7%), and CD56−CD16+ cells (2.0%).

**Figure 3:**
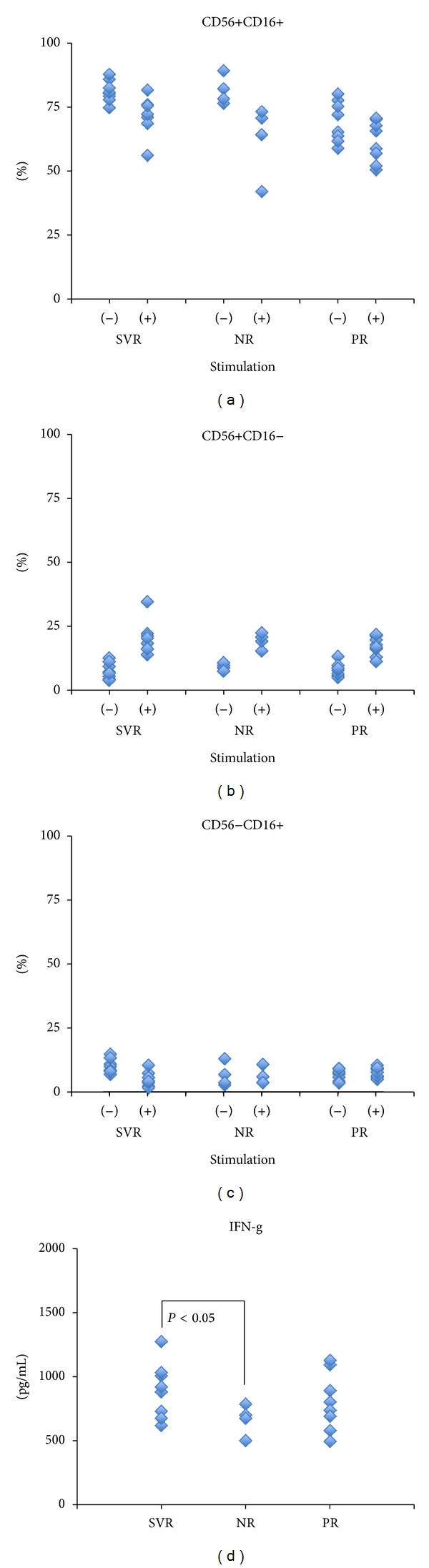
The characterization of the before and after stimulation NK cells, as classified by the SVR and NR grouping. Between (a) and (c), there were no statistically significant differences between the SVR and NR groups associated with the cell surface markers on the NK cells. However, the NK cells from the SVR group did produce higher amounts of IFN-*γ* than those from the NR group (*P* < 0.05) (d).

In order to evaluate the role of NK cells, we enriched the NK cells from the PBMC using magnetic beads. The CD3 positive fraction was completely eliminated during the isolation of the NK cells (Figure 1). The NK cells were incubated with or without LPS and IFN-*α* for 2 days, as the NK cells could not produce detectable amounts of IFN-*γ* solely with IFN-*α*. CD56 bright NK cells increased after stimulation (Figures 2(a) and 2(b)). The NK cells were classified as CD56+CD16−, CD56−CD16+, and CD56+CD16+ phenotypes (Figure 2(c)) and these phenotypes changed after stimulation (Figure 2(d)). The frequency of the CD56+CD16+ NK cells was similar in SVR patients (before stimulation: 81.1 ± 4.2%, after stimulation: 61.7 ± 8.0%), PR patients (before stimulation: 69.4 ± 7.9%, after stimulation: 71.8 ± 7.4%), and NR patients (before stimulation: 81.6 ± 5.7%, after stimulation: 62.7 ± 14.2%) (Figure 3(a)). The same tendency was observed for CD56+CD16− in SVR patients (before stimulation: 7.3 ± 3.0%, after stimulation: 19.4 ± 6.1%), PR patients (before stimulation: 7.8 ± 2.5%, after stimulation: 16.5 ± 3.6%), and NR patients (before stimulation: 10.3 ± 4.3%, after stimulation: 28.8 ± 13.7%) (Figure 3(b)) and for CD56−CD16+ in SVR patients (before stimulation: 9.5 ± 2.6%, after stimulation: 4.5 ± 2.7%), PR patients (before stimulation: 6.3 ± 2.0%, after stimulation: 7.1 ± 1.8%), and NR patients (before stimulation: 6.1 ± 4.4%, after stimulation: 5.6 ± 3.2%) (Figure 3(c)). In the CD56+CD16− fraction, the frequency was increased after stimulation regardless of the treatment response. Conversely, the IFN-*γ* production from the stimulated NK cells was higher in the SVR patients (894 ± 215 pg/mL) compared with the NR patients (668 ± 119 pg/mL; *P* < 0.05) and similar to the PR patients (804 ± 225 pg/mL) (Figure 3(d)). Prior to the IFN-*α* stimulation, the NK cells from the SVR, PR, and NR patients did not produce any detectable IFN-*γ* levels (data not shown).

### 3.3. Phenotypes and IFN-*γ* Production in NK Cells according to the *IL28B* Genotype

**Figure 4:**
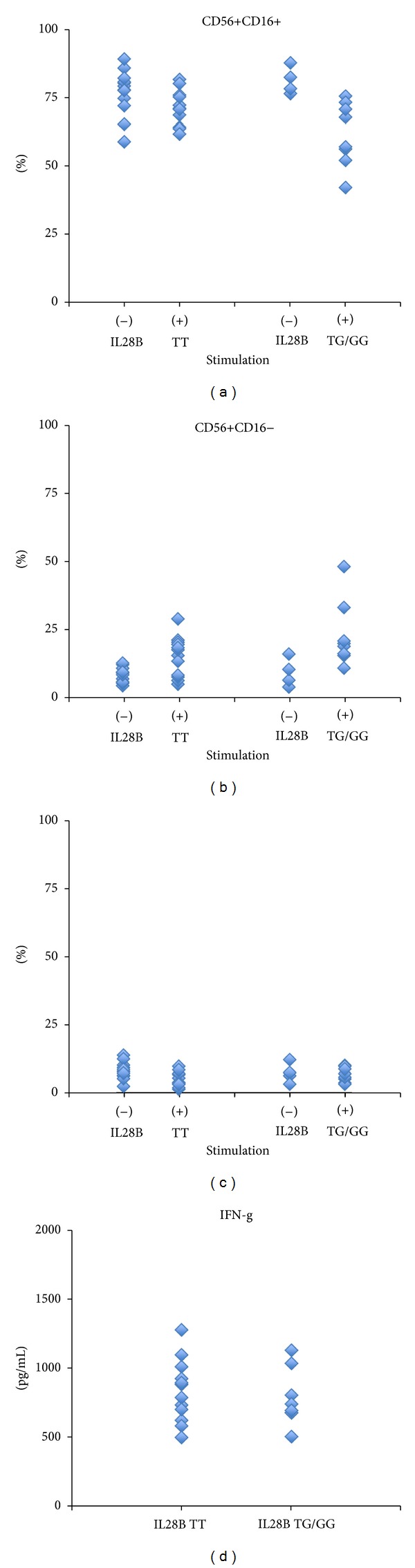
The characterization of the before and after stimulation NK cells, as classified by the *IL28B* TT and *IL28B* TG grouping. Between (a) and (c), there were no statistically significant differences noted between the *IL28B* TT and *IL28B* TG groups for the cell surface markers on the NK cells. Additionally, the NK cells from the *IL28B* TT group do not produce high amounts of IFN-*γ* when compared to the members of the *IL28B* TG group (d).

We also classified the above results according to *IL28B* genotype. The frequency of CD56+CD16+ NK cells was similar in patients with *IL28B* TT (before stimulation: 77.1 ± 8.4%, after stimulation: 71.5 ± 6.2%) and patients with* IL28B* TG (before stimulation: 71.4 ± 12.4%, after stimulation: 61.9 ± 11.8%) (Figure 4(a)). The same tendency was observed for CD56+CD16− in patients with* IL28B* TT (before stimulation: 8.2 ± 2.8%, after stimulation: 15.1 ± 7.3%) and patients with* IL28B* TG (before stimulation: 12.7 ± 5.6%, after stimulation: 22.9 ± 12.0%) (Figure 4(b)). The same tendency was observed for CD56−CD16+ in patients with* IL28B* TT (before stimulation: 8.2 ± 3.1%, after stimulation: 4.9 ± 2.6%) and patients with* IL28B* TG (before stimulation: 6.9 ± 2.7%, after stimulation: 6.7 ± 2.6%) (Figure 3(c)). Regardless of the SVR, PR, or NR classification, the IFN-*γ* production from activated NK cells was similar in patients with* IL28B* TT (833 ± 225 pg/mL) and patients with* IL28B* TG (783 ± 204 pg/mL) (Figure 4(d)).

## 4. Discussion

Because the NK cell cytotoxity and the presence of cell surface markers correlate with the virological response, NK cells can serve as biomarkers of a patient's IFN-*α* responsiveness [14, 15]. In addition, the IFN-*α*-induced modulation of the signal transducer and activator of transcription (STAT) 1 and STAT 4 phosphorylation underlies the polarization of the NK cells in an HCV infection [16–18]. During the IFN-*α* treatment, the polarized NK cells are recruited from the peripheral blood into the liver [19]. Therefore, it may be inappropriate to evaluate the peripheral NK cells for the general functions of the NK cells that are undergoing treatment. To this end, we collected the peripheral NK cells prior to treatment and evaluated the NK cell functions with IFN-*α* *ex vivo* with the aim of predicting the virological responses. Although the interactions between the killer cell Ig-like receptors (KIRs) expressed on NK cells and the HLA expressed on target cells are well known to play a key role in NK cell activation, it is difficult to obtain HCV-infected autologous target cells for the evaluation of the cytotoxity of the NK cells. Therefore, we instead evaluated the IFN-*γ* production relating to the NK cell functions.

It has been previously reported that a polarized NK cell phenotype can be induced by a chronic exposure to HCV-induced IFN-*α*, contributing to liver injury through tumor necrosis factor-related apoptosis inducing ligand (TRAIL) expression and cytotoxity [20]. Therefore, we hypothesized that we could predict an outcome of SVR or NR according to the INF-*γ* production from NK cells with or without LPS and IFN-*α* exposure. Previous reports have compared the effects of the IL-12 and IL-15 induction of NK cells, and, to this end, we prepared nonstimulated NK cells and IFN-*α* stimulated NK cells with LPS. We evaluated NK cell function in 20 patients (8 SVR, 8 PR, and 4 NR patients). The study size was small to evaluate NK cell function fully; however, we found that we could not predict an outcome of SVR according to the changes in cell surface markers, and, furthermore, the NK cell phenotype and function did not differ among the subgroups with different *IL28B* genotypes as had been previously reported [21]. These findings are consistent with the fact that NK cells do not respond to IFN-*α* via different signal transductions of type III IFN (including *IL28B*) [22, 23]. Additionally, there were no differences of this IFN-*γ* production between the patients with early virologic response (EVR) and no-EVR or rapid virologic response RVR and no-RVR (data not shown). This suggests that IFN-*γ* production from peripheral NK cells does not correlate sharply with the declining dose of HCV-RNA for the initial 2 or 4 weeks of pegylated IFN-*α* and RBV treatment.

As the NK cells kill the target cells through the directed release of perforin, granzyme-containing granules, and TRAIL which is associated with the control of an HCV infection [24], the combined effects of KIR, TRAIL, and *IL28B* in the context of IFN treatment are possible [25]. However, the evaluation of these effects would require additional studies with a larger number of patients. In addition, while the different inhibitory/activating receptors on the NK cells are well known to be associated with antiviral activity [26–29], further evaluations of the related cell surface markers must be performed.

In conclusion, these results demonstrate that the *ex vivo* NK cell response may serve as a biomarker for IFN-*α* treatment responsiveness independent of the *IL28B* genotype.

## Figures and Tables

**Table 1:** Clinical features classified by the SVR status of chronic hepatitis C patients with genotype 1.

Sex	Age (yr)	ALT (IU/L)	g-GTP (IU/L)	Plt (10^9^/L)	IL-28B (rs8099917)	Response
M	59	63	33	20	TT	SVR
M	50	31	31	20.2	TT	SVR
M	69	35	27	17.3	TT	SVR
M	31	61	25	18.5	TT	SVR
F	69	125	157	16.3	TT	SVR
M	55	66	132	18.4	TT	SVR
M	67	119	61	20.2	TG	SVR
F	52	178	101	16.1	TG	SVR
F	37	45	65	18.7	TT	NR
F	43	65	128	13.4	TT	NR
M	29	79	147	20.2	TG	NR
F	68	76	76	7.9	TG	NR
M	43	112	62	18.2	TT	PR
M	51	84	59	17.7	TT	PR
F	38	31	30	20.1	TT	PR
F	63	72	33	15.9	TT	PR
M	62	44	48	12.2	TG	PR
M	66	38	52	11.8	TG	PR
M	54	50	32	18.8	TG	PR
F	60	77	43	13.8	GG	PR
